# Computed Tomography (CT)-Assisted 3D Cephalometry in Horses: Interincisal Angulation of Clinical Crowns

**DOI:** 10.3389/fvets.2020.00434

**Published:** 2020-07-29

**Authors:** Silvio Kau, Klaus Failing, Carsten Staszyk

**Affiliations:** ^1^Department of Pathobiology, Institute of Topographic Anatomy, University of Veterinary Medicine Vienna, Vienna, Austria; ^2^Faculty of Veterinary Medicine, Institute of Veterinary-Anatomy, -Histology and -Embryology, Justus-Liebig-University Gießen, Gießen, Germany; ^3^Unit for Biomathemathics and Data Processing, Faculty of Veterinary Medicine, Justus-Liebig-University Gießen, Gießen, Germany

**Keywords:** dental imaging, equine cephalometry, incisor angle, incisor angulation, orthodontics, aging, horse

## Abstract

The angle encompassed between opposing incisors in horses is assumed to decline with age. Previous studies merely consider the overall profile view of clinical crowns presuming a generalized angle, neglecting potential tooth position-dependent differences. Cephalometric measurements from 3D computed tomographic thick-slab reconstructions of single incisors within a global reference frame were used to determine clinical crown interincisal angulation (IIA) of 48 horses. Based on predefined dentoalveolar landmarks, IIA was defined as the angle enclosed by the respective labial axis of the clinical crown (LACC). A measurement repeatability analysis was conducted including a comparison of third incisor teeth IIA with data obtained by cephalometric implementation of previously described landmarks for third incisor teeth (lingual/palatal border). The age-related angle course and differences between tooth positions were investigated considering LACCs of permanent incisors. Determining IIA by LACCs exhibited a high level of reproducibility applying for all tooth positions (mean coefficient of variation = 0.65 %; mean SD ± 0.89°). The comparison method for third incisor teeth revealed two times higher mean dispersion of repeated measurements, *P* = 0.017. A non-linear model slightly increased predictability of angular changes over time as against linearity assumption. The angle decline was more distinctive in younger horses and appears to approach a final value in older ones. Third incisor teeth exhibited significantly higher angle decline compared to first and second incisor teeth, *P* < 0.0001. According to the results, age determination of horses using clinical crown IIA is not recommended. Rather, 3D cephalometry may provide a promising tool to determine interdental and dentofacial angles of distinct tooth positions in health and disease.

## Introduction

Incisor clinical crowns have been used in many studies to investigate association of positional and morphological dental changes and the age of horses (1–6). It is widely assumed that interincisal angulation (IIA) of clinical crowns changes with increasing age. Most research papers and textbooks do not report metric angle values and use distinct angle definitions. Studies reported that based on lateral profile views or photographs in young horses the angle forms a straight or vertical line (4, 5, 7–9) and with increasing age the angle becomes more acute (2, 7, 8, 10–12), remains obtuse (4, 5) or increases (13). Those studies giving metric data suppose that in young horses the angle is about 180° (1, 3, 12, 14). Habermehl (1) described that horses between 8 and 15 years exhibit angles around 90° whereas older individuals have angles <90°. Others state that only horses > 19 years show angle values of 90° (3). Loch and Bradley (14) noticed old horses to have angles <90° but neglect to give age information. So far, IIA was specified with a single generalized angle for all incisor tooth positions. Description of methodology, however, is either subjective, subtotal, or missing.

The angle between opposing incisors was determined by a multitude of varying terms and definitions, e.g., “interincisal angle” (15), “angle between upper and lower incisors” (3–5), “angle between maxillary and mandibular incisor teeth” (16), “angle of the upper and lower incisors” (11), “direction of upper and lower incisors” (7), “incisor profile angle” (2, 12), “contact angle” (10, 14), “angle of incidence” (9, 14), or “occlusal angle” (13). Some investigators defined the angle as “the angle made by the labial borders of the upper and lower incisors” (8), “the angle formed by the labial surface of the incisive bone and lower jaw incisors” (1), “the angle between the dorsal surface of upper and lower jaw incisors” (2), or “lingual borders of the third incisor teeth” (3). In human orthodontics, the term *interincisal angle* or *angulation* is commonly used to describe the angle enclosed by opposing upper and lower jaw incisors (17–19). One recent equine cephalometric study also used this term (15). Due to a large number and sometimes confusing synonyms of most past studies in horses, we want to adapt to the established human terminology and therefore decided to use the term “interincisal angulation (IIA)” throughout the manuscript.

It is assumed that age-related positional and morphometric alterations of equine incisors may affect periodontal biomechanics (20). In contrast to brachydont teeth, equine hypsodont incisors are subjected to highly dynamic age-related morphometric and periodontal changes (21, 22). Hence, naturally occurring orthodontic forces during and after tooth eruption in horses are likely emerging to a greater extent. High orthodontic forces in humans cause complications such as alveolar bone loss or apposition and root resorption (23, 24). Arnbjerg (16) observed a higher incidence of exuberant intraalveolar cement deposition in incisors of horses which exhibit a smaller angle between opposing incisor clinical crowns. Although severity and progression of resorptive lesions in equine incisor periodontal disease appear to differ by tooth position (25), angle differences have not been examined before.

Computed tomography (CT)-assisted 3D cephalometry may present a reliable and reproducible method for defining dental landmarks and angulations at different incisor tooth positions (26, 27). Beside one cadaver head study on conventional CT based measurement of single incisor transversal and sagittal occlusal surface angles (28), no further digital 3D cephalometric studies on equine incisors are available. Domanska-Kruppa et al. (15) just recently introduced conventional two-dimensional (2D) x-ray based cephalometry to investigate class II malocclusion and IIA using distinct cephalometric landmarks in Warmblood foals. Current advances of head CT in standing sedated horses (29–31) may allow for methodical standardization as well as single tooth measurements and will make 3D cephalometry clinically applicable.

The present study aimed to establish a validated 3D cephalometric approach and to investigate age-related changes and tooth position-related differences of the IIA considering clinical crowns.

## Materials and Methods

### Animals and CT Scans

Volumetric CT datasets of heads of 950 horses were examined; however, only scans of 48 horses (age span: 2 to 20 years; mean ± SD: 8.8 ± 4.6 years) were suitable for further investigations. Exclusion criteria were: (a) horses scanned under general anesthesia, (b) ventral mandibular plane alignment not parallel to the underlying table during scanning, (c) relative horizontal jaw position not centralized, (d) image motion artifacts within the region of interest (incisors), (e) incisor or jaw fractures, (f) soft tissue interposition between incisors or (g) incisors not in contact, (h) crib-biting lesions, (i) incisor overbite, (j) overjet or underjet > half of the linguo-/palatolabial width of the occlusal table based on the previous dental examination in unsupported normal head position, (k) excessive diagonal malocclusion, and (l) horses with radiological signs of pathological incisor periodontal lesions.

**Figure 1:**
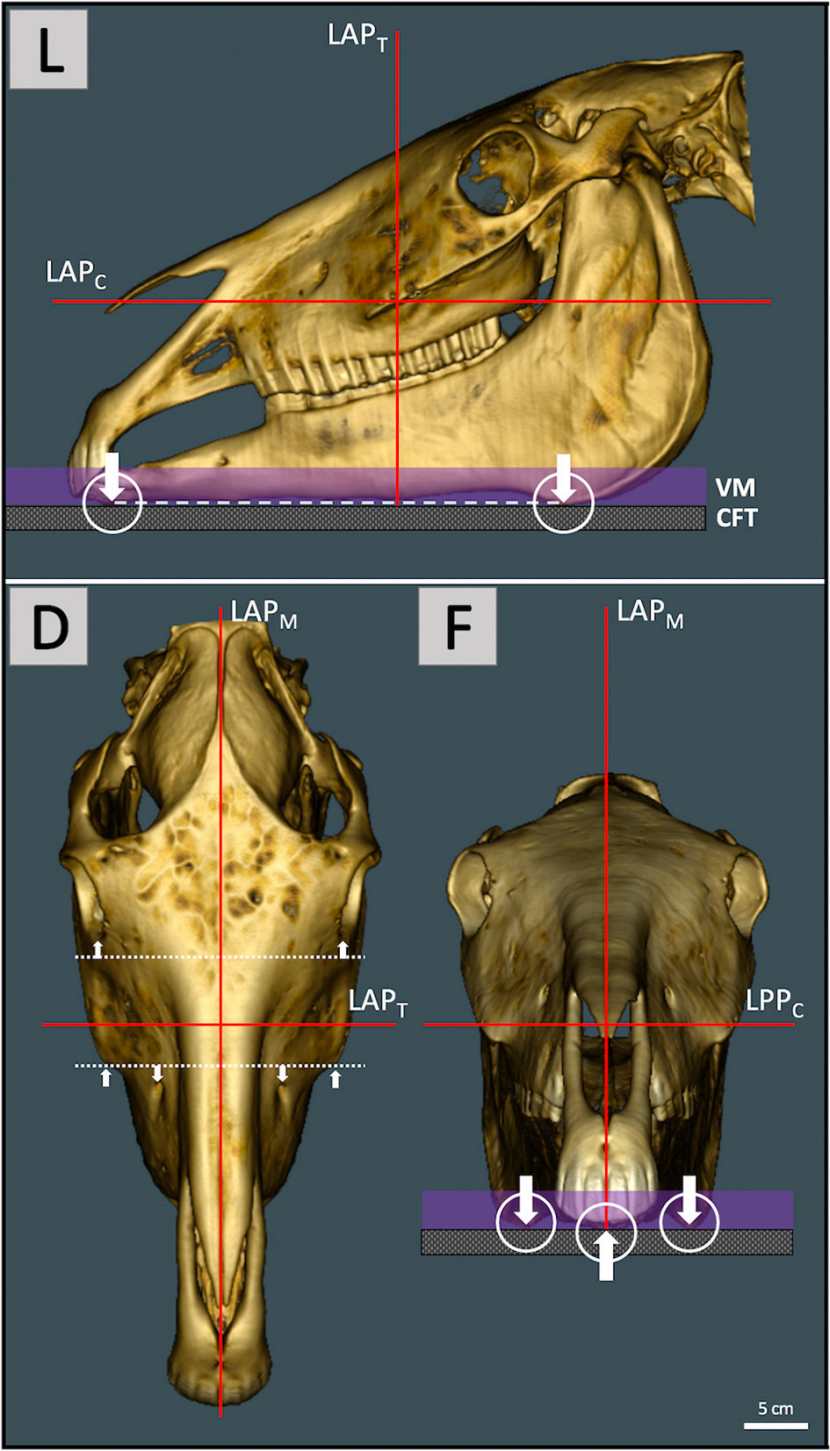
Pre-scanning laser guided orthogonal object orientation for maximal standardization of head and relative jaw position. Left lateral view (L) with the head placed on a vacuum mattress (VM) and non-absorbing levitating carbon fiber table (CFT). The ventral mandibular plane (dashed line) which connects the most ventral rostral and caudal mandibular aspects (arrows) was oriented as parallel as possible to the CFT. For optimal object centralization within the gantry opening the intersection of the transversal (LAP_T_) and coronal (LAP_C_) laser alignment plane was positioned ~3–4 cm caudal to the rostral end of the facial crest. Since the laser patient positioning system is static, the horse and CFT had to be moved up and down. (D) The dorsal view shows orientation of the median laser alignment plane (LAP_M_) along the median plane of the head and orthogonal LAP_T_ preferably parallel to adjacent symmetry axes (dotted lines, arrows). In the frontal view (F) ventral mandibular aspects should not exhibit rotation along a rostrocaudal axis.

Included animals were 26 female (54.2%) and 22 male horses (45.8%), 44 large breeds (esp. Warmbloods and Quarter horses) and four small breeds (two Haflinger, Arab Mix, and Riding pony). Head CT scans were obtained from horses referred to the Veterinary Clinic Gessertshausen, Germany, during 2009–2017 due to dental, maxillofacial, or sinonasal disorders limited to areas caudal to upper and lower jaw bars. A multislice helical Siemens Somatom Sensation 16 or Somatom Sensation Open CT scanner (Siemens Healthcare, Forchheim, Germany) was used as a sliding gantry with the horses standing. Horses were sedated with 0.01 mg^*^kg^−1^ detomidine hydrochloride (Vetoquinol, Ismaning, Germany) and positioned and fixed for standing head CT examination as described by (29). Laser guided orthogonal object orientation and height adjustment of both the examination-stand and underlying carbon fiber table had been used to ensure positioning of the head as standardized as possible (Figure 1).

The setting of the CT scanner was 140 kV and 180 mAs. Based on the topogram, x-ray source current was automatically modulated suitable to individual size and anatomy of the head using Siemens CARE Dose4D real-time radiation exposure control. Scan sections with a single slice thickness of 0.6 mm, and greyscale values according to tissue specific x-ray beam attenuation were generated.

### 3D Reconstruction of Single Incisor Teeth

**Figure 2:**
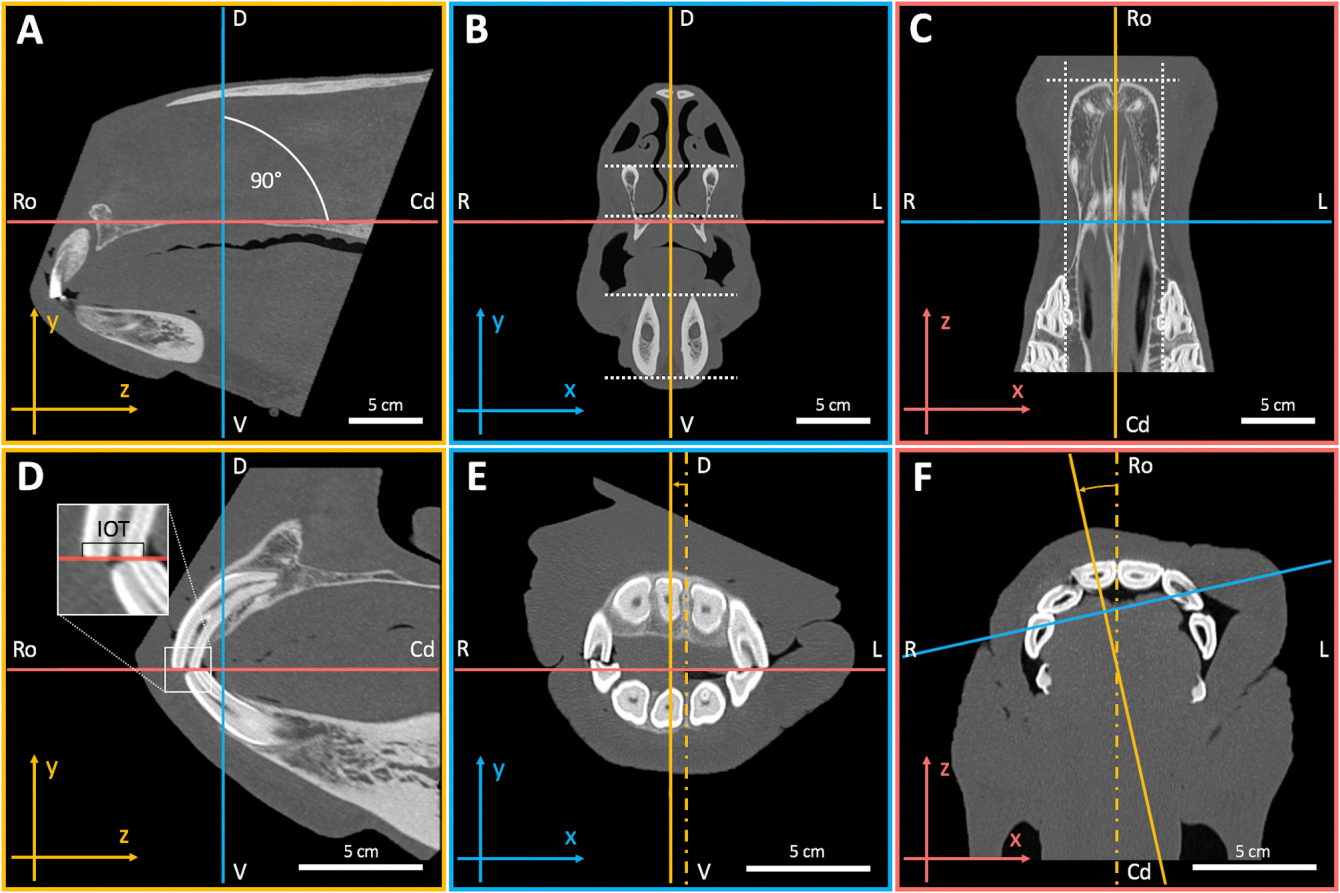
Definition of a 3D virtual global reference frame (GRF) using multiplanar reconstruction (MPR) and the two-dimensional (2D) on-screen Cartesian coordinate system (CCS). MPR adds a third dimension (z-axis) to the x- and y-axis of the 2D CCS (corresponds to the x- and y-axis in b). The axes were set orthogonal (90° offset) in all three dimensions, which resulted in a sagittal **(A,D)**, transversal **(B,E)**, and coronal **(C,F)** section plane view. Initially the axes were rotated until the sagittal z-axis **(A)** and transversal **(B)** and coronal x-axes **(C)** matched the horizontal x-axis of the on-screen CCS. The sagittal axes (orange) were orthogonal to this **(B,C)**. For maximal standardization of object orientation, the object was first aligned along the axes of the transversal **(B)** and coronal **(C)** section planes using some symmetry planes (dotted lines). Subsequently, the region of interest has been enlarged [**(D–F)**, zoom factor 1.7]. The sagittal axes (orange) were translationally moved off from the median plane to the right side **(E)** and rotated **(F)** until the tooth 101 appeared in the sagittal plane view **(D)**. There the object was rotated along a laterolateral axis until the incisor occlusal table (IOT; see insert) hits the coronal z-axis (red). Object alignment was rechecked. To create a GRF for single tooth reconstruction, the object was then spatially fixed within the coordinate system by avoiding rotation and translation of the object as well as rotation of orthogonal axes in the sagittal and transversal view. R, right; L, left; D, dorsal; V, ventral, Ro, rostral; Cd, caudal.

**Figure 3:**
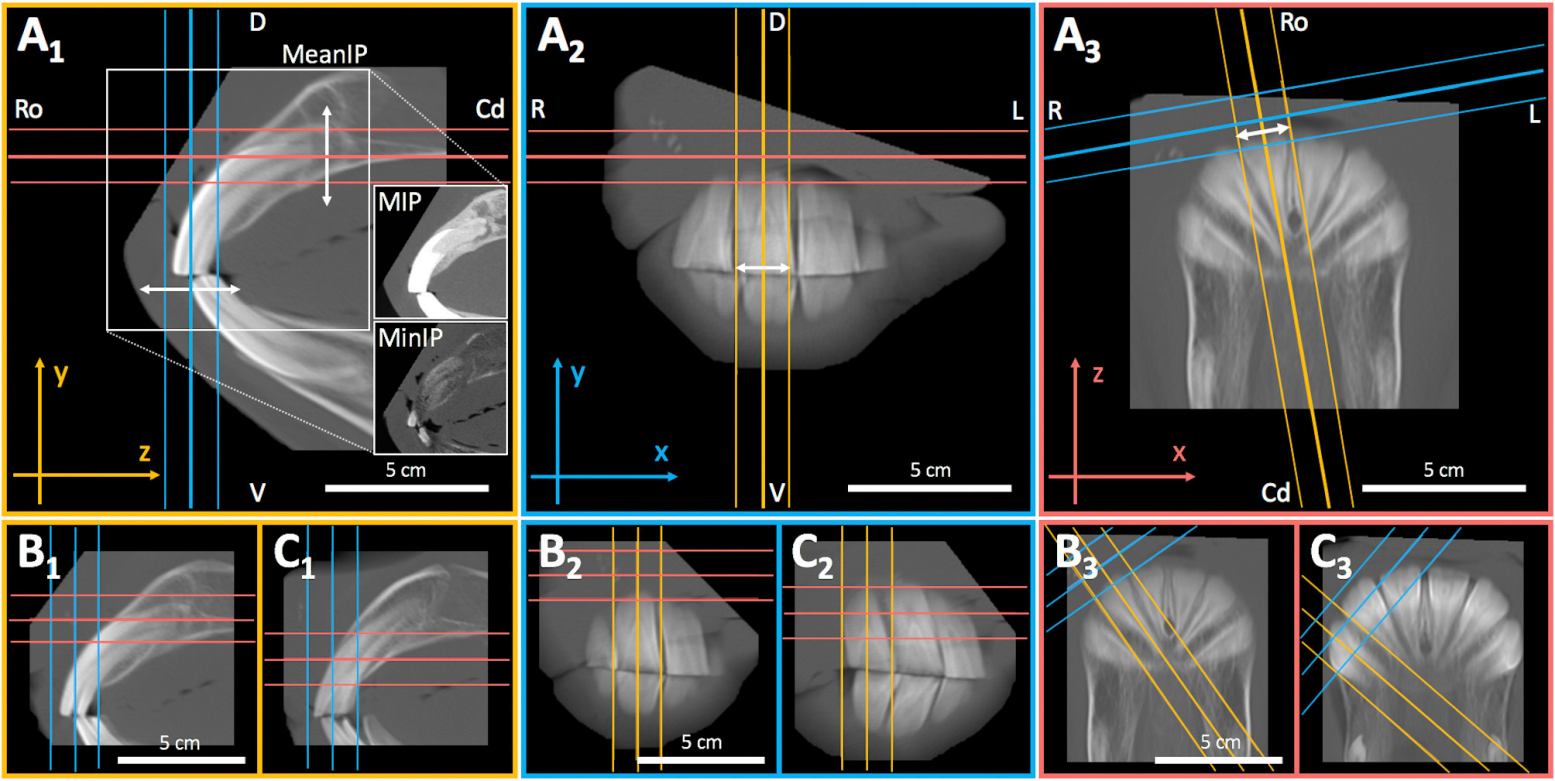
3D multiplanar thick slab reconstruction (TSR) of individual incisors. TSR was used to merge contiguous slices within a certain range of the scan volume, hence creating a 2D x-ray-like sagittal image of each tooth. Superpositions with adjacent dental tissue structures could thus be reduced to a minimum. **(A**_**1**_**)** For optimal quality of TSRs the digital image calculation mode was set to “mean intensity projection” (MeanIP). Compare to the modalities maximum- (MIP) and minimum intensity projection (MinIP). **(A–C**_**2−3**_**)** Thick slab width was chosen according to the maximal mesiodistal tooth width of each single incisor (arrows) which was determined by running through the transverse and coronal plane of the tooth [arrows in **(A**_**1**_**)**]. The GRF allowed translational axis movements in the sagittal and transversal view **(A–C**_**1−2**_**)** as well as translational and rotatory axis movements in the coronal view **(A–C**_**3**_**)**. The axes have been adjusted to reconstruct the tooth in its entire sagittal apicoocclusal extend without axial distortion **(A–C**_**1**_**)**. Repeating this for each upper jaw incisor (B = 102, C = 103) and opposing lower jaw incisors resulted in 12 sagittal DICOM images per horse. R, right; L, left; D, dorsal; V, ventral, Ro, rostral; Cd, caudal.

Digital imaging and communications in medicine (DICOM) files were imported to the open source medical image viewer Horos (version 2.2.0, The Horos Project^1^). Greyscale values were set to a window width of 3,100 and window level of 500 HU for optimal visualization of mineralized tissues. Multiplanar reconstruction (MPR) mode and orthogonal axis orientation were used to virtually align and spatially fix the object, creating a 3D virtual global reference frame (GRF) for upper and lower jaw facial subunits (Figure 2). The GRF allows sagittal reconstruction of upper and lower jaw incisors without changing their 3D positional relationship that is mandatory for assessing IIA (Figure 3). The modified Triadan system was used for numbering of different tooth positions (32, 33).

### Post-reconstruction Definition of Incisor Axes and IIA Measurement

**Figure 4:**
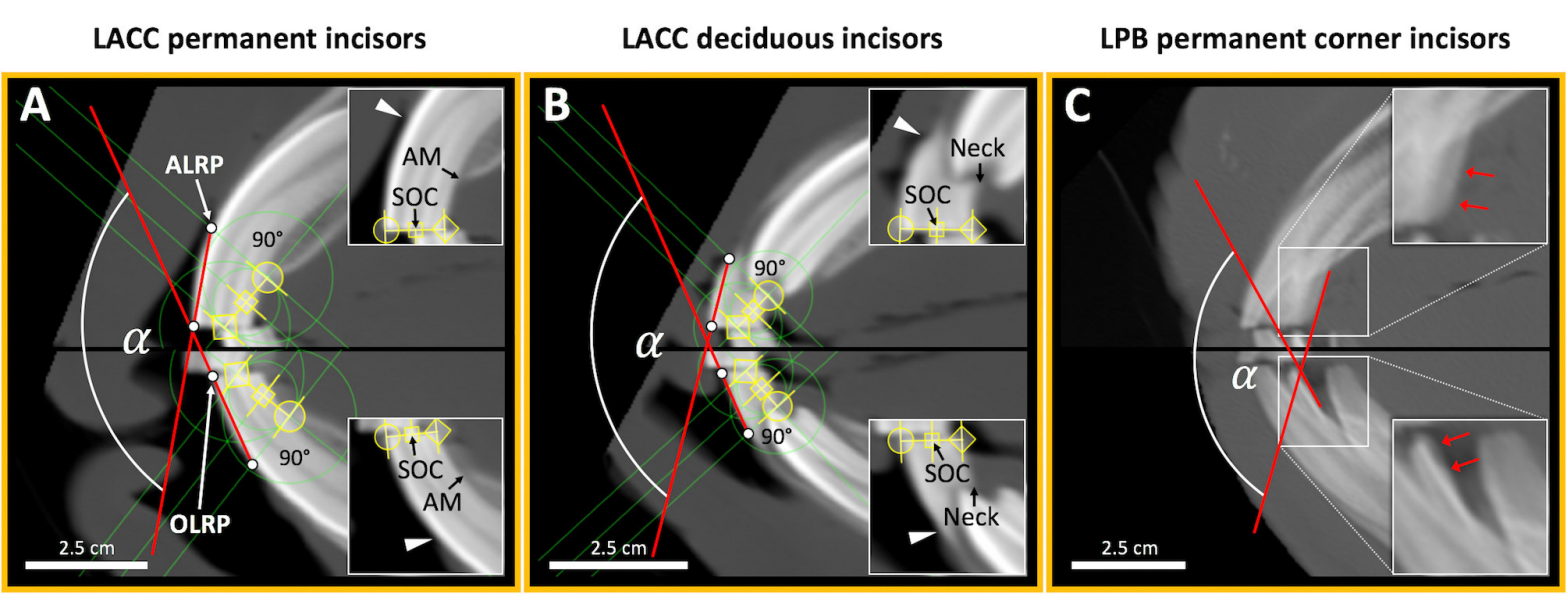
Determination of IIA by cephalometric definition of the labial axis of opposing permanent and deciduous incisor clinical crowns (LACC) and the lingual and palatal clinical crown border reference axes (LPB) for permanent third incisor teeth. **(A,B)** LACC reconstructions required definition of certain dental landmarks starting with the sagittal occlusal center (SOC). The SOC was determined by bisecting the occlusal surface of individual incisors (inserts). For that purpose, PixelStick endpoints (yellow) were positioned to the most labial (circle endpoint) and palatal or lingual (diamond endpoint) occlusal points. The SOC was thus automatically ascertained by the ruler instrument. Subsequently, endpoints were positioned to connect the SOC and palatal or lingual alveolar margin (AM) of permanent incisors and palatal or lingual crown neck of deciduous incisors (inserts). A line perpendicular to the SOC-AM line and the AM point, in turn, defined the apicolabial reference point (ALRP) at the intersection with the labial tooth surface. For scans that reveal the gingival margin (inserts; arrow heads), the ALRP always appeared close or superposed to it. The respective occlusolabial reference point (OLRP) was defined as the most labial occlusal point. Connection of ALRP and OLRP resulted in the respective LACC (red line) for each incisor. IIA was defined as the angle enclosed by the LACC of opposing incisors. **(C)** The angle enclosed by the adopted lingual and palatal reference axes of third incisor teeth was determined (red lines; arrows). All angles were measured using the angle measuring capabilities of PixelStick. A, 101:401; B, 501:801; C, 103:403; labial, left; lingual or palatal, right.

The 2D DICOM images of sagittal reconstructed incisors were exported to a tagged image file format (TIFF). The common GRF ensures that reconstructed sagittal images of opposing deciduous or permanent incisors have the original *in vivo* positional relationship. Images were retrieved within the 2D on-screen CCS and edited using the image editing capabilities of the program PowerPoint (Microsoft Corporation, Redmond, WA, US). The sagittal image of each tooth was used to define dentoalveolar landmarks for cephalometric reconstruction of the respective labial axis of the clinical crown (LACC) (Figures 4A,B). A previous photograph-based study used the lingual and palatal border (LPB) of third incisor teeth clinical crowns to assess overall IIA (3). We adopted these landmarks to cephalometrically implement them for direct comparison with LACC reconstructions of third incisor teeth (Figure 4C). IIA of third incisor teeth using LACC and LPB reconstructions was compared in all horses randomly assigned to the measuring repeatability analysis. Landmarks, tooth axes, and IIA were determined using the orthogonal axis orientation and length and angle measuring capabilities of the open source on-screen ruler PixelStick (version 1.2.1, Plum Amazing LLC., Princeville, Hawaii^2^). PixelStick software also uses the on-screen CCS.

### Determination of Measurement Repeatability

From all horses included in the study (*n* = 48) a randomized selection of 10 horses (*n* = 10), of which five (*n* = 5) were older than 12 years, was made. The IIA between the LACC of opposing incisors 101:401, 102:402, 103:403 was measured. Additionally, IIA between the LPB of 103:403 was determined and compared to values obtained from LACC measures. The angle between each pair of predefined opposing incisors was measured 10 times (*n* = 10). The blinded measurements took place on 10 (*n* = 10) different days at two-day intervals. Block randomization was used to determine the order in which horses were examined on respective days (Supplementary Material). The daily results were transferred to another spreadsheet so that values were not accessible to the observer (SK) on following days.

### Statistical Analysis

Analyses were performed using the statistical software package BMDP (programs 1D, 6D, 5V, AR; Dixon, W.J., University of California, LA, USA), open source R software (version 3.6.1; used function: lmer from the R library lme4; R Foundation for Statistical Computing, Vienna, Austria^3^), SPSS software (version 24.0; IBM Analytics, NY, USA), and Microsoft Excel (version 2016; Microsoft Corp., WA, USA). Univariate descriptive statistics was used to describe central tendency and dispersion of IIA data from both the deciduous and permanent incisors.

The measurement variability was investigated using variance decomposition separated by tooth position (01-03) and measuring method (LACC, LPB). Measurement repeatability was further characterized calculating the coefficient of variation (CV) according to horse, tooth position, and measuring method. CV was defined as SD/mean^*^100 (%), respectively. A student's *t*-test for paired samples was used to compare LACC vs. LPB with respect to the CV and mean maximum difference (Diff_max_) of repeated IIA measurements on third incisor teeth. Concerning LACC, differences in the CV and Diff_max_ between tooth positions were compared using one-way repeated measures ANOVA.

Due to few observations and the short period in which deciduous incisors occur, these were only considered descriptively. Strength of linear association between IIA measures of corresponding permanent incisor tooth positions of the right and left side and between the respective IIA (dependent variable) and the age of horses (independent variable) was analyzed using linear regression and correlation analysis. A Pearson's correlation coefficient (*r*) of |*r*| < 0.30 was suggested to indicate negligible correlation; |*r*| = 0.31–0.50 low positive or negative correlation; |*r*| = 0.51–0.70 moderate positive or negative correlation; |*r*| = 0.71–0.90 high positive or negative correlation and |*r*| ≥ 0.91 meant very high positive or negative correlation (34). The coefficient of determination (*r*-squared) was calculated as *r*^2^.

To produce improved parameter estimates, the relationship of IIA of permanent incisors (dependent variables) and the age of horses (independent variable) was analyzed allowing non-linearity using derivative-free non-linear regression analysis. The fitted model equation is described as

$$\begin{array}{l} f\left(age\right)=E+A*exp\left(-b*age\right) \end{array}$$

where *E* denotes the final value of the course for a very high age value, *A* the amplitude and *b* the exponential factor of decline. Due to the iterative character of the method the initial parameter values were: *E* = 100, *A* = 60, *b* = 0.25. The coefficient of determination (pseudo-R^2^) was calculated as *1-(sum of squared residuals/corrected sum of squares)*. For examination of the discrepancies between observed and predicted values (model goodness-of-fit) observed vs. predicted data plots and residual analysis metamodels including residuals vs. predicted plots, residuals vs. order plots, residual vs. age plots, and Q-Q-plots were made.

Global side-, tooth position-, and age-related effects and side-by-tooth position-interaction were approximated statistically using two-factorial incomplete repeated measures analysis of covariance (ANCOVA) with an asymptotic maximum likelihood approach and Wald test of significance. The side (left, right) and tooth position (01-03) were entered the analyses as fixed effects. The age of horses was entered as a covariate and IIA as the dependent variable. To account for individual variation between investigated horses, these were entered into the analyses as a random effect. The Akaike information criterion (AIC) was used to compare data fits and to select an appropriate covariance structure.

The influence of age on the angle differences between different tooth positions (age-by-tooth position-interaction) was analyzed using a generalized linear mixed-effect (GLME) model with a restricted maximum likelihood approach for estimation of covariance and Wald test of significance. The values of corresponding tooth positions on both sides were summarized. The tooth position was considered as a fixed effect, the age as a covariate, IIA as the respective dependent variable, and both individual horses and the side were treated as random effects. Residuals were graphically inspected for normal distribution using Q-Q-plots.

For all statistical tests performed, a *p* ≤ 0.05 (significance level) was considered to indicate statistical significance.

## Results

A total number of 950 individual CT scans was checked for exclusion criteria. There were 125 scans (13.2%) that displayed the region of interest, but only 48 met the inclusion criteria (38.4%). The main exclusion reasons were motion artifacts (24.8%), non-standardized head position (16.8%), missing or supernumerary incisors (7.2%), and rostral jaw fractures (5.6%). Thus, 576 (*n* = 576) incisors were individually reconstructed.

Three horses (6.3%; 2.3 ± 0.6 years) exhibited full deciduous incisor dentition. Five horses (10.4%; 4.2 ± 0.5 years) showed only third incisor teeth to be deciduous, whereas permanent first and second incisor teeth already featured full occlusal contact. The newly described cephalometric measuring approach (LACC) could be applied to all incisors. Regardless of the tooth position, tooth generation, or age, the clear majority of examined horses showed an obtuse IIA of LACCs (> 90° < 180°).

### Measurement Repeatability Analysis

The randomly selected horses younger than 12 years (*n* = 5) showed a mean age (± SD) of 7 ± 2.9 years (span: 4–11) whereas older horses (*n* = 5) showed a mean age (± SD) of 17 ± 2.6 years (span: 14–20). Except for one horse, repeated IIA measures resulted in a total of 40 angular values which corresponds to 10 independent measurements per tooth position and measuring method. Due to irregular and short palatal clinical crown border of the deciduous tooth 503 in horse 1, IIA using LPB reconstructions could not be determined resulting in a total of 30 angular values (Supplementary Material). Descriptive statistics are presented in Table 1. Comparing LACC and LPB reconstructions on third incisor teeth, IIA revealed a mean angle difference (± SD) of 9.0° ± 7.0°. In nine horses (90%) LACC reconstructions constantly yielded higher values (Min = 2.2°, Max = 21.8°). Only one horse (10%, Warmblood, age = 11 years) exhibited higher IIA obtained from LPB reconstructions (6.4° ± 1.3°, Min = 4.1°, Max = 8.4°).

**Table 1:** Descriptive statistics and relative variability for repeated cephalometric interincisal angulation measurements by horse, tooth position, and measuring method.

	**Method**								
**Horse**	**LACC**	**LPB**	**Mean (α)**	**Variance**	**SD (α)**	**Min (α)**	**Max (α)**	**Diff_**max**_ (α)**	**CV (%)^**a**^**
	**Tooth position**								
1	01	-	153.9	0.72	0.85	152.8	155.3	2.6	0.55
	02	-	149.3	0.38	0.62	148.4	150.5	2.0	0.42
	03	-	122.5	2.74	1.66	118.1	123.6	5.5	1.35
	-	03^b^	-	-	-	-	-	-	-
2	01	-	147.8	0.36	0.60	146.4	148.6	2.2	0.41
	02	-	145.2	0.68	0.82	144.0	146.9	3.0	0.57
	03	-	148.9	0.65	0.81	148.0	150.2	2.2	0.54
	-	03	131.3	9.74	3.12	126.2	134.1	7.8	2.38
3	01	-	139.5	0.61	0.78	138.3	140.9	2.6	0.56
	02	-	135.8	0.76	0.87	134.3	137.7	3.4	0.64
	03	-	120.0	0.26	0.51	119.0	120.7	1.8	0.43
	-	03	112.0	2.86	1.69	109.7	115.0	5.3	1.51
4	01	-	131.6	0.27	0.52	130.5	132.5	2.0	0.40
	02	-	130.6	0.40	0.63	129.6	131.6	2.1	0.48
	03	-	124.0	0.33	0.58	123.1	124.8	1.7	0.47
	-	03	118.1	1.83	1.35	116.4	120.5	4.1	1.14
5	01	-	137.3	0.98	0.99	135.9	139.3	3.4	0.72
	02	-	131.5	0.54	0.73	130.3	132.9	2.6	0.56
	03	-	122.3	0.07	0.27	122.0	122.9	0.9	0.22
	-	03	128.7	1.78	1.33	126.6	131.3	4.7	1.04
6	01	-	147.2	1.01	1.00	145.4	149.0	3.6	0.68
	02	-	140.1	0.29	0.54	139.2	141.2	2.0	0.39
	03	-	116.1	0.97	0.98	115.1	118.0	2.9	0.85
	-	03	111.3	0.67	0.82	109.9	112.9	3.1	0.74
7	01	-	110.3	0.26	0.51	109.6	111.1	1.5	0.46
	02	-	109.1	0.50	0.71	108.0	110.4	2.4	0.65
	03	-	87.5	0.12	0.35	86.7	88.0	1.2	0.40
	-	03	72.1	1.40	1.18	70.4	74.7	4.3	1.64
8	01	-	121.0	0.24	0.49	120.1	121.5	1.4	0.41
	02	-	122.7	0.30	0.55	122.0	123.5	1.5	0.45
	03	-	113.8	1.32	1.15	112.2	116.0	3.8	1.01
	-	03	100.7	1.26	1.12	98.7	102.2	3.5	1.11
9	01	-	120.0	2.84	1.68	117.6	123.8	6.1	1.40
	02	-	120.9	1.45	1.20	119.6	123.9	4.3	1.00
	03	-	108.9	1.11	1.05	107.2	110.5	3.3	0.97
	-	03	98.1	2.38	1.54	95.4	100.6	5.2	1.57
10	01	-	118.9	0.96	0.98	117.0	119.6	2.6	0.83
	02	-	112.9	0.43	0.66	111.8	114.1	2.3	0.58
	03	-	101.8	1.99	1.41	98.9	103.6	4.7	1.38
	-	03	90.4	1.14	1.07	88.5	91.5	2.9	1.18

*LACC, labial axis of the clinical crown; LPB, lingual and palatal border; α, alpha angle degrees; SD, standard deviation; Min, minimum; Max, maximum; Diff_max_, maximum difference; CV, coefficient of variation; Values are based on 10 (n = 10) repeated measurements for each tooth position or measuring method*.

a *CV represents the SD in percent of the mean*.

b *The short and irregular lingual and palatal surface of the deciduous upper jaw incisor clinical crown did not allow accurate definition of a clinical crown axis and, hence, no determination of the IIA*.

**Figure 5:**
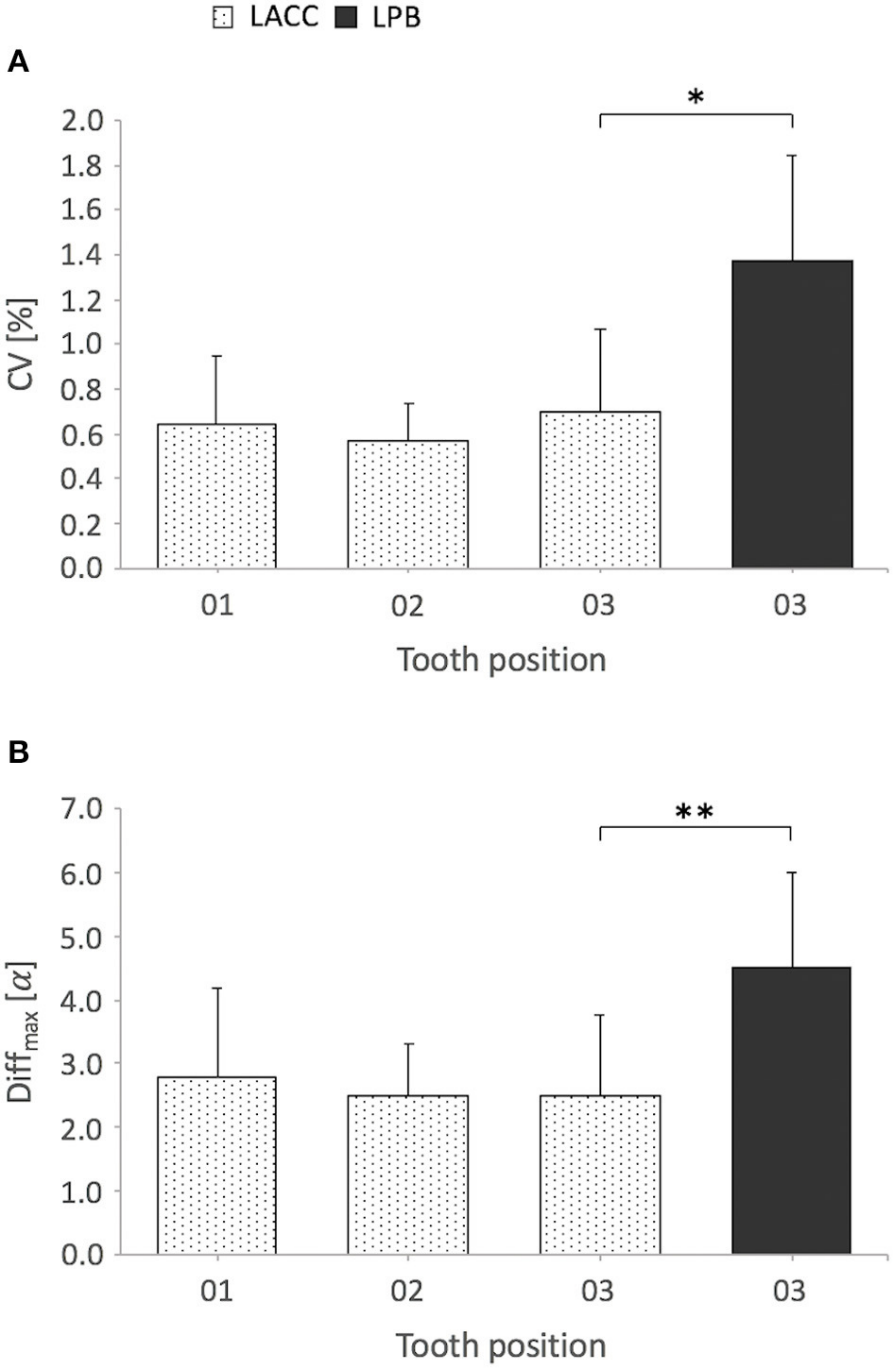
Mean coefficient of variation (CV) and mean maximum angular difference (Diff_max_) of repeated interincisal angulation measurements by measuring method and tooth position. Bar plots display mean + SD (error bars); α, alpha angle degrees. Cephalometric determination of the angle encompassed by the respective labial axis of the clinical crown (LACC) of opposing third incisor teeth exhibited a significantly lower CV **(A)** and Diff_max_ **(B)** compared to the reference method that uses reconstruction of the lingual and palatal border (LPB); *P = 0.017, **P = 0.030, Student's *t*-test for paired samples, *n* = 9 horses (missing LBP observation horse 1). Comparing tooth positions 01-03 for LACC reconstructions, there was no significant difference in the CV **(A)** and Diff_max_ **(B)**: CV, *P* = 0.312, Diff_max_, *P* = 0.860, one-way repeated measures ANOVA, *n* = 10 horses (LACC observation horse 1 included), sphericity assumed.

Variance decomposition of repeated measurements and resulting standard deviation for different tooth positions and measuring methods and throughout tooth positions (LACC) are shown in Table 2. The minimum angle standard deviation resulting from the calculated minimum variance of LACC reconstructions was ± 0.27° whereas the maximum variance revealed a maximum standard deviation of ± 1.68°. The minimum SD resulting from the calculated minimum variance of LPB reconstructions was ± 0.82° whereas the maximum variance revealed a maximum SD of ± 3.12° which is 1.9 times higher than that using LACC reconstructions. Independent of the scaling level all measured data showed <2.4% dispersion around the respective mean of repeated measurements (CV range = 2.16%). However, using LACC for IIA measurements resulted in significantly higher precision and lower maximum angle differences compared to LPB reconstructions (Figure 5). Measurement precision and Diff_max_ did not significantly differ between tooth positions concerning LACC reconstructions (Figure 5).

**Table 2:** Variance decomposition and resulting mean angle standard deviation (SD [α]) for repeated cephalometric interincisal angulation measurements by measuring method, tooth position, and across tooth positions for LACC reconstruction.

**Method**	**Tooth position**	**Mean variance**	**Min variance**	**Max variance**	**SD (α)**
LACC	01	0.83	0.24	2.84	0.91
	02	0.57	0.29	1.45	0.76
	03	0.96	0.07	2.74	0.98
	All	0.79	0.07	2.84	0.89
LPB	03	2.56	0.67	9.74	1.60

*LACC, labial axis of the clinical crown; LPB, lingual and palatal border; α, alpha angle degrees; Min, minimum; Max, maximum; Values are based on n = 100 repeated measurements per tooth position for LACC reconstructions and n = 90 for LPB reconstructions*.

### Linear and Non-linear Regression Analysis

**Figure 6:**
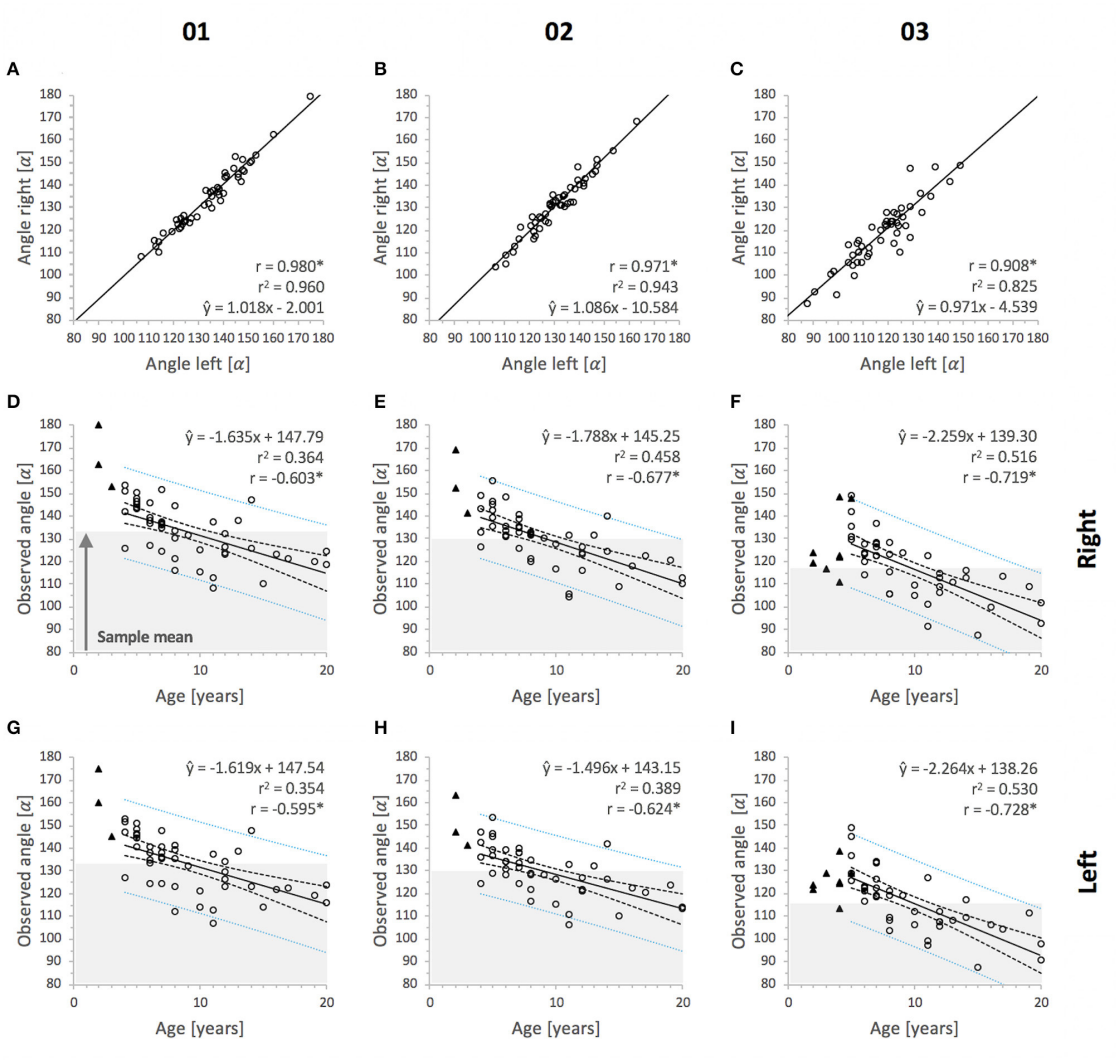
Linear regression and Pearson's correlation analysis show the association of observed IIA values between both sides and the age of horses. The prediction equation and regression parameters are displayed in the plots and specified as ŷ_*i*_ = ± β_*1*_ *(slope) x*_*i*_ ± β_*0*_ *(intercept)*; α, alpha angle degrees. **(A–C)** Correlation diagrams show the association of IIA between corresponding permanent and deciduous incisors from the left and right side. Pearson's correlation coefficients (*r*) and coefficients of determination (*r*^2^), displayed in the plots, were calculated for each tooth position: **(A)** Triadan 01, **(B)** Triadan 02, **(C)** Triadan 03. *Positive correlations were significant at *P* < 0.001 (two-sided), *n* = 48 horses. Line indicates trend line. **(D–I)** Correlation diagrams show the association between IIA of permanent incisors and the age of horses. The upper limit of gray areas indicates mean values for permanent incisors. IIA data from deciduous incisors (triangles) were not included in the analysis. *r* and *r*^2^ were calculated for each tooth position and displayed in the plots. *Negative correlations were significant at *P* < 0.001 (two-sided), *n* = 45 horses (01 and 02), *n* = 40 horses (03). Lines indicate trend lines; inner dashed lines indicate upper and lower 95% confidence intervals for the regression line and outer dotted lines indicate upper and lower 95% prediction intervals.

Further analyses were based on the extended data set and respective LACC reconstructions. The descriptive statistics summarizing IIA values of both the deciduous and permanent incisors and the age of investigated horses are shown in Table 3. Comparison of observed IIA values from corresponding deciduous and permanent incisors of both sides revealed a very high positive linear correlation (Figures 6A–C). A moderate to lower high negative linear correlation was calculated comparing observed IIA values of permanent incisors and the age of horses (Figures 6D–I). Pearson's correlation coefficients (*r*), coefficients of determination (*r-squared*), and associated *p*-values are given within the graphs. Contemplating data scatter and from a biological point of view, however, IIA may not decrease infinitely even in horses older than 20 years. Though, the angle decrease was additionally checked for non-linearity.

**Table 3:** Descriptive statistics of observed interincisal angulation (IIA) using LACC from deciduous (D) and permanent incisors (P) and age distribution of all examined horses by tooth position and side.

**Side**	**Tooth position**	***N***	**IIA (α)**					**Age (years)**			
			**Mean**	**SD**	**Min**	**Max**	**Range**	**Mean**	**SD**	**Min**	**Max**
R	D 01	3	165.1	13.85	152.8	180.1	27.3	2.3	0.6	2.0	3.0
R	D 02	3	154.0	13.89	141.2	168.8	27.6	2.3	0.6	2.0	3.0
R	D 03	8	126.5	14.04	110.9	148.5	37.6	3.5	1.1	2.0	5.0
L	D 01	3	160.0	14.91	145.0	174.8	29.8	2.3	0.6	2.0	3.0
L	D 02	3	150.4	11.16	141.5	162.9	21.4	2.3	0.6	2.0	3.0
L	D 03	8	125.5	7.26	113.3	138.9	25.5	3.5	1.1	2.0	5.0
R	P 01	45	132.7	12.08	108.5	153.9	45.4	9.2	4.5	4.0	20.0
R	P 02	45	129.7	11.78	104.2	155.5	51.3	9.2	4.5	4.0	20.0
R	P 03	40	117.0	13.60	87.5	148.9	61.4	9.9	4.3	5.0	20.0
L	P 01	45	132.6	12.13	107.3	153.2	45.9	9.2	4.5	4.0	20.0
L	P 02	45	129.3	10.68	106.4	153.6	47.2	9.2	4.5	4.0	20.0
L	P 03	40	115.9	13.46	87.8	148.7	60.9	9.9	4.3	5.0	20.0

*R, right; L, left; α, alpha angle degrees; SD, standard deviation; Min, minimum; Max, maximum*.

**Figure 7:**
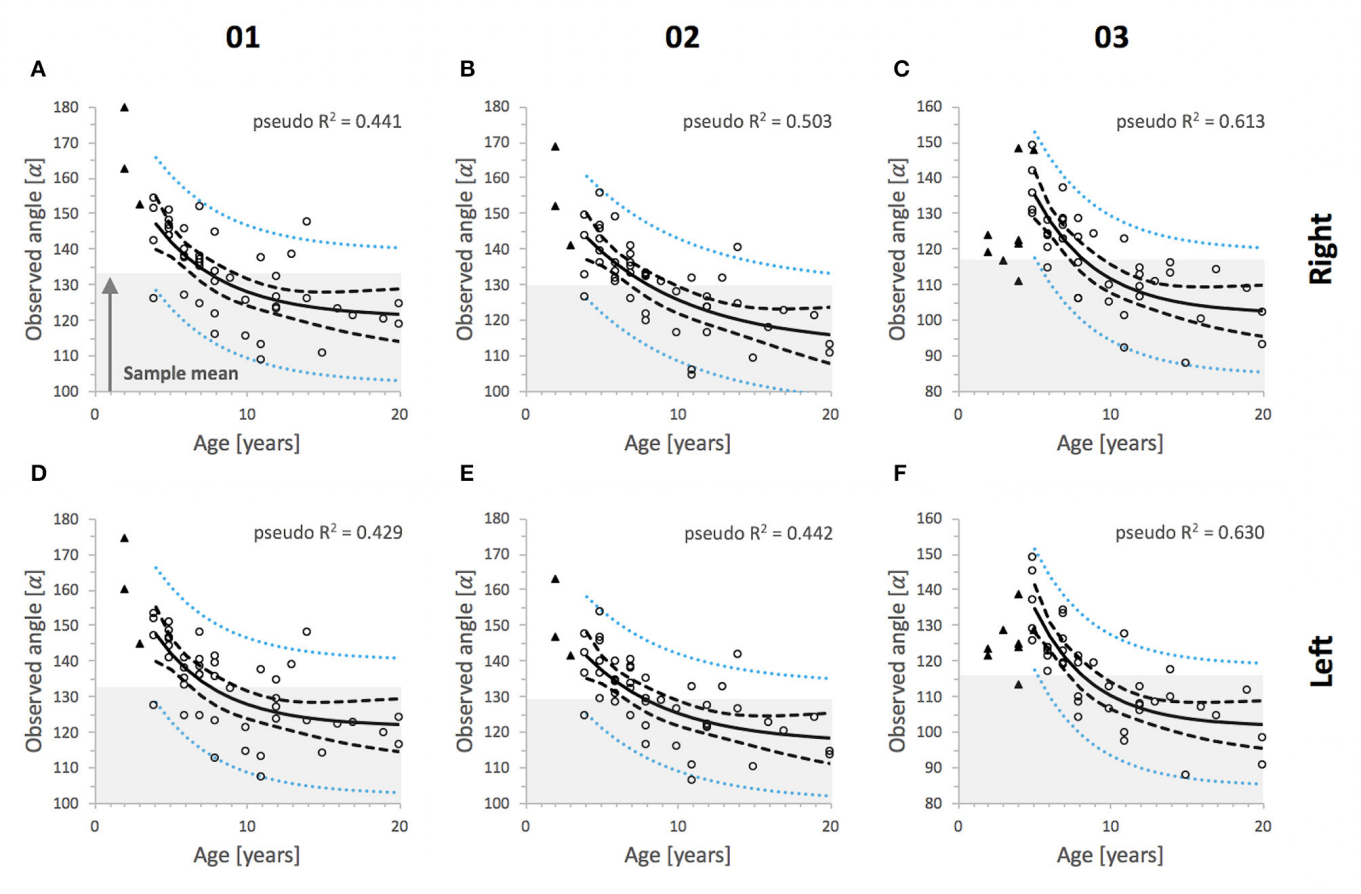
Non-linear regression analysis shows the association of observed IIA values and the age of horses by tooth position and side. **(A–C)** right side, **(D–F)** left side; α, alpha angle degrees. The upper limit of gray areas indicates mean values for permanent incisors. IIA data from deciduous incisors (triangles) were not included in the analysis. Pseudo-*R*^2^ was calculated for each tooth position and displayed in the plots, *n* = 45 horses (01 and 02), *n* = 40 horses (03). Lines indicate trend lines; inner dashed lines indicate upper and lower 95% confidence intervals for the non-linear regression line and outer dotted lines indicate upper and lower 95% prediction intervals.

Time-dependent prediction of IIA using non-linear regression analysis was shown to provide slightly more accurate estimates for the course of angular decrease compared to linear regression analysis (Figure 7). Calculated minimum residual sum of squares and model parameters (*E, A, b*) are displayed in Table 4. Estimated values for *E* in permanent dentition shared low CV indicating high precision of estimate whereas due to mathematical reasons this is not the case for estimated total amplitude (*A*) and exponential factor of angle decline (*b*). Goodness-of-fit analysis revealed an appropriate fit of the applied non-linear regression model. The residuals appeared normally and randomly distributed and exhibited a constant variance which indicates unbiased parameter estimates (Supplementary Material). The angle decline appears more distinctive in younger compared to older horses, where the curve approaches a hypothetical final value (*E*). All tooth positions from both sides showed a slightly higher coefficient of determination (pseudo-*R*^2^) in non-linear regression (Figure 7). An additional 7.45 ± 2.23% of the observed variation in the response variable (IIA) was explained by the model. The third incisor teeth showed the highest pseudo-*R*^2^ and most distinctive non-linearity of angle decline (Figures 7C,F).

**Table 4:** Model parameter estimates that were returned by tooth position and side using an iterative non-linear least squares procedure using the regression function *f(age)* = *E* + A*exp(-b*age).

			**Parameter**								
**Side**	**Tooth position**	**Minimum residual sum of squares**	***E*(α)**	**SE**	**CV(%)**	***A*(α)**	**SE**	**CV(%)**	***b***	**SE**	**CV(%)**
R	01	3589.5	120.7	5.1	4.2	63.4	26.3	41.5	0.22	0.11	51.8
R	02	3038.5	112.8	8.2	7.3	54.2	11.9	22.0	0.14	0.09	59.1
R	03	2787.7	102.1	4.6	4.5	112.9	49.2	43.6	0.25	0.10	38.8
L	01	3706.1	121.4	4.7	3.9	67.4	29.9	44.4	0.24	0.12	49.5
L	02	2800.5	117.1	5.8	4.9	49.9	16.5	33.1	0.18	0.10	58.2
L	03	2616.3	101.8	4.1	4.0	125.0	57.1	45.7	0.27	0.10	36.1

*R, right; L, left; α, alpha angle degrees; E, final value of the course; A, amplitude; b, exponential factor of decline; SE, standard error; CV, coefficient of variation. Values are based on n = 45 horses for first (01) and second incisor teeth (02) and n = 40 horses for third incisor teeth (03)*.

Although it showed a slightly more distinct angle decrease in younger horses and not infinite angle decrease in older horses, the overall linear regression model appears suitable indicating predictive power in the population. Thus, to avoid changes in the error structures and interpretation of inferential results by data transformation, marginal inaccuracies between the non-linear and linear models were accepted in the approximation of angle differences and the influence of age.

### Side-, Tooth Position-, and Age-related Effects

The inferential statistical analyses considered *n* = 40 complete cases (full permanent incisor dentition) and *n* = 5 cases with missing dependent variables (deciduous third incisor teeth) whereas *n* = 3 cases were excluded from the analyses due to full deciduous incisor dentition.

Global comparison of IIA from corresponding tooth positions of the left and right sides revealed no significant difference (Wald chi-squared = 1.0730, *P* = 0.300). It was observed that 4.2% of horses (*n* = 2) exhibited equal IIA of either corresponding first or third incisor teeth, whereas no horse revealed angular symmetry in second incisor teeth. In most horses, however, slight angular asymmetry was present. Compared to corresponding left incisors, 50.0% of remaining horses exhibited higher IIA values in right first incisor teeth, 56.3% in right second, and 60.4% in right third incisor teeth. The mean difference (± SD) in first incisor teeth was 5.1° ± 2.7° for deciduous and 2.1° ± 1.6° for permanent teeth, 3.8° ± 3.0° and 2.7° ± 1.8° in second incisor teeth, 8.9° ± 6.1° and 3.6° ± 2.5° in third incisor teeth.

The ANCOVA and associated Wald test were indicative of an overall significant angle difference between distinct permanent incisor tooth positions (Wald chi-squared = 591.05, *P* < 0.0001). The analysis of the interaction side-by-tooth position showed that the side (left vs. right) has no influence on observed differences between tooth positions (Wald chi-squared = 0.617, *P* = 0.735). The age of horses, however, was shown to has a significant global impact on angle changes (Wald chi-squared = 41.19, *P* < 0.0001). The combined average angle decrease was −1.8° per year.

**Figure 8:**
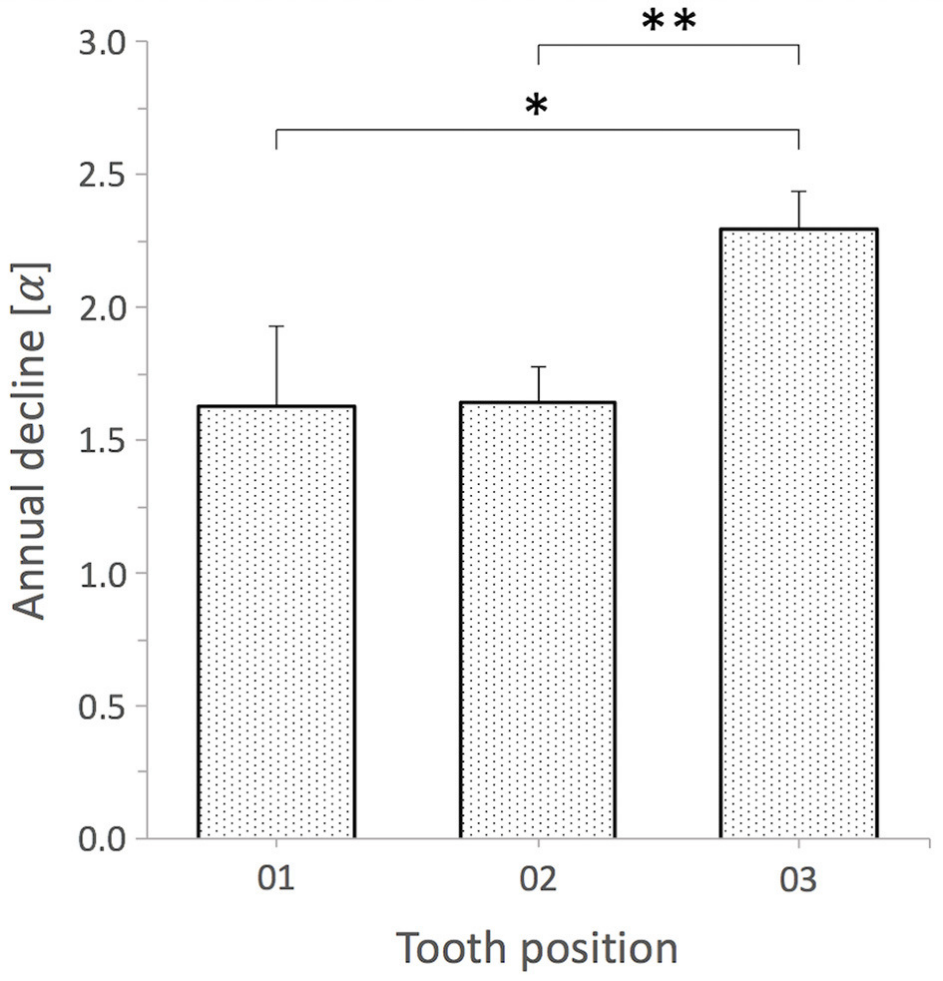
Comparison of annual angle decline of permanent incisors by tooth position. Bar plots display mean + SE (error bars). Tooth position 01 vs. 03 and 02 vs. 03 exhibit significant differences; *, **P < 0.0001 whereas tooth position 01 vs. 02 did not; *P* = 0.915, GLME model, *n* = 45 horses (01 and 02), *n* = 40 horses (03).

Further analyzes using the GLME model combine values obtained from corresponding tooth positions on both sides. The comparison of the angle declines of individual tooth positions as a function of time (age-by-tooth position) showed a significant difference on average (Wald chi-squared = 26.99, *P* < 0.0001). The respective average degree of annual angle decreases of first and second incisor teeth showed hardly any differences, whereby third incisor teeth stand out with a stronger decline (Figure 8). Over time, 72.2% of first incisor teeth exhibited larger IIA than second incisor teeth. Over time, 95.5% of first and second incisor teeth exhibited larger IIA than third incisor teeth.

## Discussion

The present study was designed to establish a validated 3D cephalometric approach to measure the angulation between opposing incisor clinical crowns identifying age and tooth position related effects. A measurement repeatability analysis was conducted to assess precision of both the newly established (LACC) and adopted (LPB) measurement methods. To evaluate IIA of clinical crowns some prior studies used lateral profile photographs of equine incisors (2–4, 7) and others merely considered profile views (1, 5, 16). However, all of which did not describe exact measuring methodology or anatomical landmarks and considered IIA as a single generalized angle value for all opposing tooth positions. Muylle et al. (3) described that just the lingual borders of third incisor teeth clinical crowns were used to determine IIA. However, the data report suggests that the angle was also treated under generalization in this study. Domanska-Kruppa et al. (15) implemented lateral 2D cephalograms to assess IIA of upper and lower jaw first incisor teeth in foals. This study comprehensively describes used dentofacial cephalometric landmarks and resultant incisor reference lines, the superior and inferior incisal line, which intersection was considered to specify IIA in horses aged up to 12 months. Undoubtedly, this study makes a valuable contribution to orthodontic measurements of class II malocclusions in horses. This 2D method, however, does not apply to investigate all incisor tooth positions independently and reference lines do not exclusively relate to clinical crowns or functional and anatomical incisor longitudinal axes. Thus, a method was needed that avoids superimposition of anatomical structures enabling clear landmark identification and IIA measurements of all left and right incisor tooth positions separately.

CT-assisted 3D cephalometry provides a highly reliable and reproducible method for dental angular measurements (26). In humans, its applications may allow for separate reconstruction, landmark identification, and angle measurements of all incisor tooth positions, particularly in complex orthodontic anomalies like crowded incisors (26, 27). Comparison of 2D vs. 3D cephalometry in human orthodontics showed significant differences of upper incisor dentofacial inclination and interincisal angulation (27, 35). Jodeh et al. (35) recommend selective decision of the method to maximize measuring accuracy in distinct cephalometric questions and advice caution when measuring incisor angles. In 2D, the IIA is interpreted as the pitch between opposing incisor axes whereas 3D axis reconstructions within a global coordinate system account for pitch, roll, and yaw directional parameters all codetermining the angle (36). Thus, 2D projection of 3D structures as well as superimposition of adjacent anatomical structures of the left and right side (e.g., large incisors in horses) and head position during scanning must be considered as major limiting factors in 2D cephalometry (37). Both, low contrast and resolution of obtained images may additionally influence landmark identification (26).

The primary CT reconstruction algorithm creates a volume of data that is subdivided into regularly shaped volume elements (voxels), each assigned a 3D Cartesian coordinate system. The gray value of each voxel is associated with the x-ray beam attenuation of the represented object/tissues (38). Under certain conditions, a smaller voxel size may lead to a higher spatial resolution. The multislice CT scans used here create voxels larger in size compared to those may obtained by cone beam CT. In a study in which multislice CT and cone beam CT were compared, however, Watanabe et al. (39) showed that a smaller voxel size does not always improve spatial resolution as this may also depend on both the in-plane and longitudinal directed modulation-transfer function. Nevertheless, cone beam CT has not yet become area-wide established in equine dentistry due to various limitations, such as substantial sensitivity to motion artifacts, higher scatter, or a limited dimension of the field of view (FOV) (31). Additional artifacts of cone beam CT are alias artifacts by x-ray beam divergence and a higher noise level (38). In general, the stacked voxels representing the object volume can be displayed in any direction thus eliminate projection geometry error and superposition of adjacent structures. In the present study, the unique 3D alignment of different tooth positions was considered with the aid of CT-assisted multiplanar thick slab reconstruction in the acquisition of X-ray-like 2D images of individual incisors by orthogonally synthesizing associated voxel information within a global reference frame. It should be noted that the method presented in our study, in contrast to landmark identification and angle measurements on reconstructed 3D volumes (27), uses 3D image reconstruction and 2D landmark identification and measurements. It is important to mention that thick slab reconstructions may introduce another source of error by superimposing the mesial side of the incisor over the middle and distal sides. Thus, thick slab reconstructions are somewhat limited identifying exact labial or lingual/palatal clinical crown surfaces. This ambiguity would increase if the direction of reconstructed 2D longitudinal sections was misaligned. However, this approach also brings about a reduction of superimpositions with large neighboring incisors to a minimum and avoids object enlargement and distortion which is a result inherent to conventional 2D x-ray projections of craniofacial structures (40).

The rotation of the head was described as one of the leading factors causing regional image magnification and distortion in human 2D cephalometry, both promoting measurement inaccuracy (40, 41). Even with 3D cephalometry a precise measurement of the IIA of opposing upper and lower jaw incisors requires consideration of 3D relative jaw mobility and resulting spatial dislocation of opposing incisors during CT scanning. It should be considered that the relative rostrocaudal mobility of the mandible to the upper jaw may depend on the head position (42). The sole rostrocaudal translational jaw movement would not alter the angular positional relationship of opposing incisors. However, we know little about rotatory degrees of freedom of the mandible along a laterolateral, rostrocaudal, and dorsoventral axis, which may alter the angular positional relationship of opposing incisors at different head positions. Thus, it is recommended to ensure a head position that is as standardized as possible during the scans. Domanska-Kruppa et al. (15) used a custom made cephalostat to guarantee the best possible standardization of head position during acquisition of lateral 2D cephalograms in foals. We recently reported and show in the present study how the head position of horses can be kept as standardized as possible considering technical and safety aspects during standing head CT scans (29). In future studies it may be possible to implement a cephalostat for safe use during head CT in standing sedated horses. Hence, no scans that were performed under general anesthesia or scans that do not fulfill requirements for maximal standardization of the head position were used in the current study. A metal tube in between incisors, which is commonly used to protect the tracheal tube during general anesthesia in horses and resulting occlusal forces, may cause a tilting movement of incisors. Schrock et al. (20) had shown slight tilting movement of equine incisors in a finite element model when the occlusal surface is loaded with physiological occlusal forces. This could influence reliability of IIA measurements.

The lingual and palatal border (LPB) of third incisor teeth clinical crowns were suggested to be reproducible reference structures due to their rough surface straightness. Thus, we additionally used these to determine alternative IIA measurements on third incisor teeth. Although repeated IIA measurements using LPB exhibited low dispersion around the sample mean, landmark-based LACC reconstructions from the newly implemented 3D cephalometric method educed higher precision (SD ± 1.60° vs. 0.98°) and two times lower dispersion. Independent of the scaling level the mean SD for all tooth positions using LACC was 0.89° which may be acceptable for clinical and scientific use. A previous study in humans had shown that there is hardly any difference between intra- and interobserver variability of repeated angle measurements (<0.65°) using either 2D or 3D cephalometry (27). However, reproducibility of landmark identification is a basic requirement in human cephalometric angle and linear measurements (41). Since the reference axes have merely been aligned along the LPB of third incisor teeth, observed surface curvature and irregularity could have caused the angle measurement inaccuracy. It might be also harder to repeatable align axes along with a short surface structure. Both in humans and horses, repeated linear cephalometric measurements exhibit highest errors for short distances (15, 41). The computerized sagittal incisor reconstructions reported here were suitable to obtain defined dentoalveolar landmarks for labial tooth axis definition. Similar to a previous study (15) the most labial occlusal point was used as an occlusal reference point. Contrary, they used the incisive bone cusp and mandibular alveolar process cusp as a second more apical reference point whereas we used an apicolabial reference point approximating the gingival margin. Hence, the first cephalometric IIA measurements of clinical crowns under standardized conditions became possible.

In other publications, the reference structures described to determine IIA of clinical crowns, such as the labial surface of the incisive bone and lower incisors (1), labial border (8), or dorsal surface of upper and lower jaw incisors (2), however, are difficult to understand from a basic differential geometrical standpoint. Equine incisors display a convexly curved labial surface (6, 22), which according to own investigations (data not shown) is more pronounced in first and second than in third incisor teeth and changes with advancing age. A note of caution is due here since on a curved surface each arbitrary point has its own tangent. This in turn will result in different values for the clinical crown IIA depending on which tangent of upper and lower jaw incisor labial surface is chosen. It is not clear from previous studies which dental landmarks were used to define the axes for measurement of permanent incisor clinical crown IIA. The methodical approach of the present study allowed clear dentoalveolar landmark identification and IIA measurements of single incisor tooth positions with high precision. This is a basic requirement to contribute to a thorough understanding of naturally occurring orthodontic forces and periodontal disease in the highly dynamic and adaptive incisors of horses.

Equine hypsodont teeth are subjected to continuous occlusal wear, thus age-related morphometric adaptation and permanent periodontal remodeling is required (21, 22, 43). It was assumed that age-related dental angular changes and tooth length have a pertinent role contributing to the development of incisor periodontal disease in horses (20). Up to 4 years after eruption, equine permanent incisors compensate occlusal wear by apical new formation of dental hard tissues but exhibit a determinate increase in length from this time point. Interestingly, this maximum length can be maintained up to an approximated dental age of 13 to 15 years, whereat total tooth length differs by the tooth position or jaw (22). Thereafter, occlusal wear is no longer compensated by new formation of dental substances resulting in a shortening of intraalveolar tooth length and, thus, reduction of the periodontal ligament attachment surface (6, 21, 43). Non-linear regression analysis revealed that the period of greatest decrease in clinical crown IIA coincides with that of the reported constant tooth length ratios (<13–15 years). The third incisor teeth showed an initially stronger decrease in clinical crown IIA. In contrast, during the period of subsequent tooth length decrease (> 13–15 years) the angle decline had a significantly smaller negative slope and tend to approach an end value. These findings are virtually contrary to previous studies which have suggested that a decrease of the angle encompassed between upper and lower jaw incisors is accompanied by a shortening of incisor length (1, 9, 44). Although our results are based on few observations in older horses a low CV in the calculation of final values (*E*) indicates high precision of estimate.

Symptoms and progression of EOTRH, which is a periodontal disease mainly affecting incisors of aged horses (45), tend to differ by tooth position (25). Therefore, assessing angulation of pairs of opposing incisors was intended. The side (left, right) exhibited no influence on angle differences whereas age did. The GLME model provided evidence for a higher overall annual angle decline of third incisor teeth compared to first and second incisor teeth. The latter shared no significant angle differences. It may be that third incisor teeth are probably the most susceptible teeth to naturally occurring orthodontic forces due to their exposed position in the incisor arch. Although third incisor teeth are by tooth age the youngest of the incisors, they most often show clinical and radiographic signs of EOTRH (25, 46). Henry et al. (25) reported external replacement resorption as the most prevalent type of resorptive lesions in EOTRH syndrome progressing from third to first incisor teeth with an increasing manner. Contrary, external inflammatory resorption which is the second most prevalent type of resorption in EOTRH affected horses' progresses from first to third incisor teeth. Both replacement and inflammatory resorption, however, are subjected to completely different etiologies in humans. It is assumed that in most cases both types of resorption are preceded by mechanical injury (orthodontic pressure) to non-mineralized tissues initiating the resorption process (47). In addition to the formation of cementum-like repair tissue, the progression and type of resorption depend on the further stimuli, e.g., pressure or infection. Whether resorption progresses or cement is deposited may depend on the extent of damage caused by the initial intraalveolar injuries (47). Arnbjerg (16) observed a higher incidence for exuberant intraalveolar cement deposition of incisors from horses which exhibit a more acute angle between opposing incisor clinical crowns. This was suggested to be caused by altered distribution of occlusal stresses. Schrock et al. (20) demonstrated in a finite element incisor model that with increasing age of horses at constant occlusal force loading periodontal stress distribution changes with higher forces appearing in regions of first radiological signs of EOTRH lesions. This combination of findings supports the conceptual premise that mechanical stimuli, likely differing by tooth position, may be of great etiological importance in the EOTRH syndrome complex. Interestingly, Kunz et al. (48) reported that they have found no signs of EOTRH in 70 Brazilian working horses, aged 18 ± 4 years, which have never had any dental treatment. How dental treatment, tooth curvature, and direction and rate of tooth eruption affect IIA changes remains unclear. However, it should be considered that our results relate to angular measurements of clinical crowns only. To draw further conclusions on incisor periodontal disease, the examination of angle changes considering incisors in their whole apicoocclusal extend is indicated for both healthy and EOTRH diseased horses.

## Study Limitations

The present study is confined to a small number of horses representing a convenience sample experimental design as no longitudinal data were available; hence, results should be interpreted cautiously. Due to general cost issues and the mostly poor periodontal status of at least more than one incisor, no CT scans of fully “incisor-healthy” horses aged over 20 years were available. Due to practical constraints, this paper only provides intraobserver variability assessment of repeated cephalometric measurements. The paper does not engage with measurements of EOTRH horses.

## Conclusion

The purpose of the current study was to establish a validated CT-assisted 3D cephalometric approach to measure IIA of clinical crowns from all opposing incisors at lowest level of superimposition with adjacent structures and to determine age and tooth position related effects. This study has found high repeatability of dentoalveolar landmark identification and subsequent IIA measurements using the newly implemented cephalometric method (LACC; mean SD ± 0.89°). Despite the few observations, a non-linear age-related angle decline, most distinctive in third incisor teeth, was shown. The IIA tends to approach an end value in older horses. Comparison of tooth positions revealed that third incisor teeth show a significantly higher overall angle decrease than first and second incisor teeth. Tooth position–related differences of IIA suggest a role for biomechanical alterations promoting equine incisor periodontal disease. An age estimate based on the IIA of the clinical crowns is not recommended. The authors recommend that in future studies a comprehensive description of the imaging technique, anatomical landmarks, and measurement method, as well as a correct geometric definition of the angle types are indispensable. In addition, more emphasis should be placed on using correct cephalometric terminology.

## Data Availability Statement

The raw data supporting the conclusions of this article will be made available by the authors, without undue reservation.

## Ethics Statement

Ethical review and approval for this study was not required according to national legislation because all investigated computed tomography data were obtained for clinical reasons not related to this study. Informed consent has been granted by the owners after supplied with sufficient information regarding care and diagnostic and treatment options for their animals. The use of achieved diagnostic material complied with the guidelines specified by the local ethics committees of the University of Veterinary Medicine Vienna and the Justus Liebig University Gießen to be based on the regulations of good scientific practice.

## Author Contributions

SK and CS conceived of the presented idea and designed the study with the help of KF. CS and KF supervised the work, contributed to the interpretation of the results, and worked on the manuscript. SK conducted all tooth and axis reconstructions and did the measurements. SK and KF performed the computations and verified the analytical methods. SK wrote the main manuscript with the support of CS and KF. SK prepared all figures and tables with input from CS and KF. All authors provided critical feedback and revised and reviewed the final manuscript.

## Conflict of Interest

The authors declare that the research was conducted in the absence of any commercial or financial relationships that could be construed as a potential conflict of interest.

## Footnotes

^1^See: https://horosproject.org/download-horos.

^2^See: https://plumamazing.com/product/pixelstick.

^3^See: https://www.r-project.org.
