# Supplementary material for: Computed Tomography (CT)-Assisted 3D Cephalometry in Horses: Interincisal Angulation of Clinical Crowns
Source: Front Vet Sci. 2020 Jul 29;7:434. doi: 10.3389/fvets.2020.00434 (PMC7403475; doi:10.3389/fvets.2020.00434)
Supplement: Supplementary file 1 [file Data_Sheet_1.PDF]

Supplementary Figure 1. Goodness-of-fit-analysis of the nonlinear regression model (A-E) and normal distribution testing of residuals obtained with the GLME model (F).

A

Observed vs predicted plots by side and tooth position

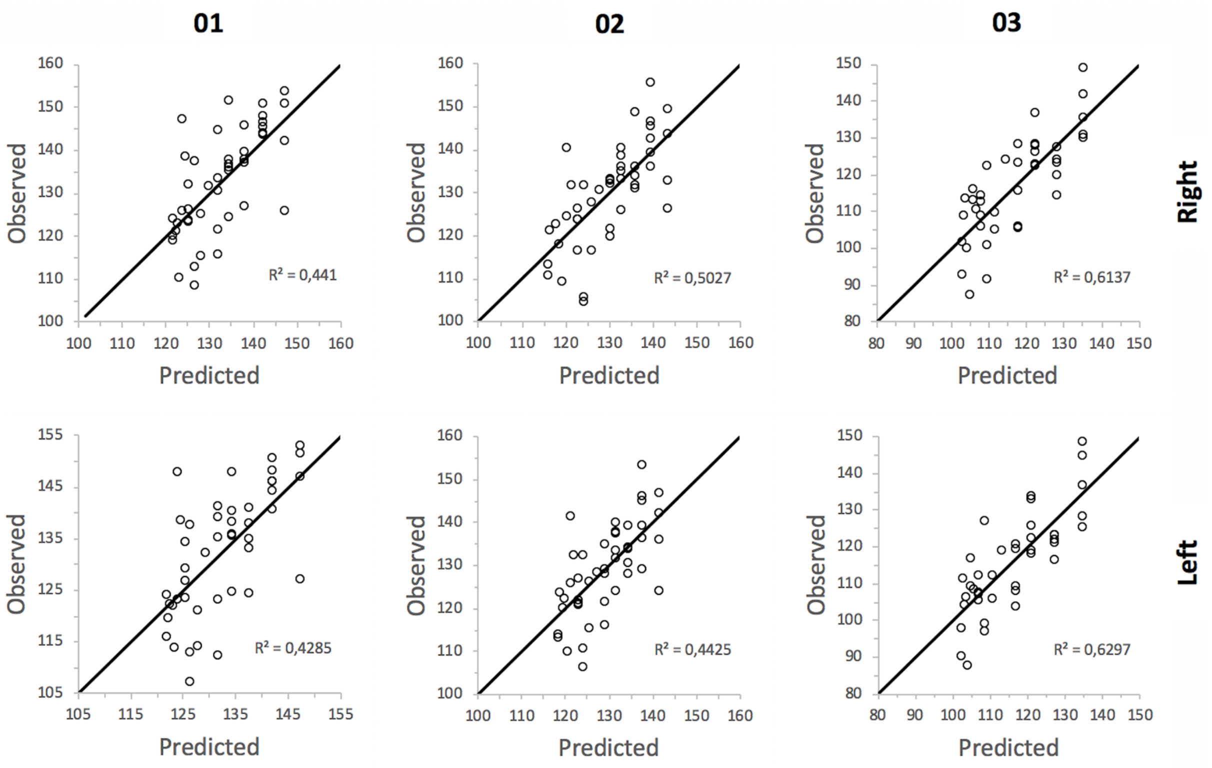

There is an upper moderate to high correlation between the model's IIA predictions (angle degrees) and its observed IIA values (angle degrees) indicating acceptable accuracy of the nonlinear regression model.

B

Residuals vs predicted plots by side and tooth position

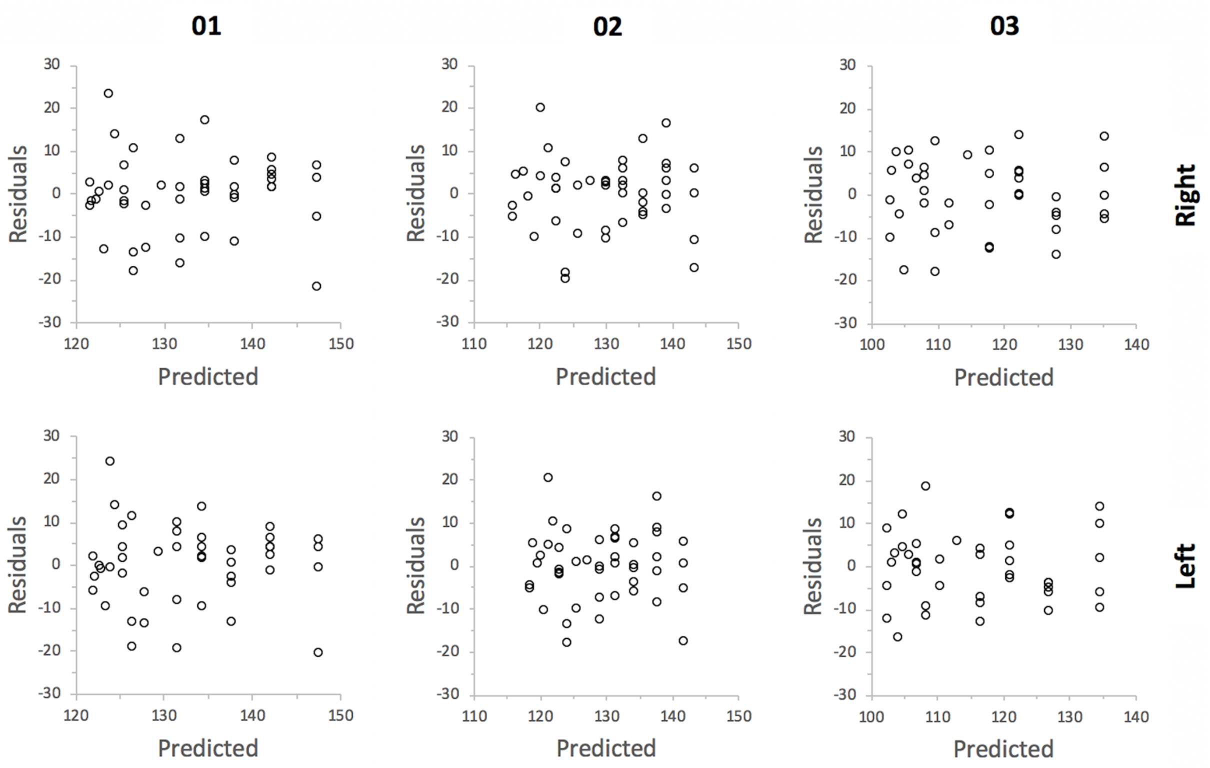

The residuals (angle degrees) bounce randomly around the zero-line indicating unbiased parameter estimates respectively equal variances of error terms along the regression line (homoscedasticity). Predicted IIA values (angle degrees).

C

Residuals vs order plots by side and tooth position

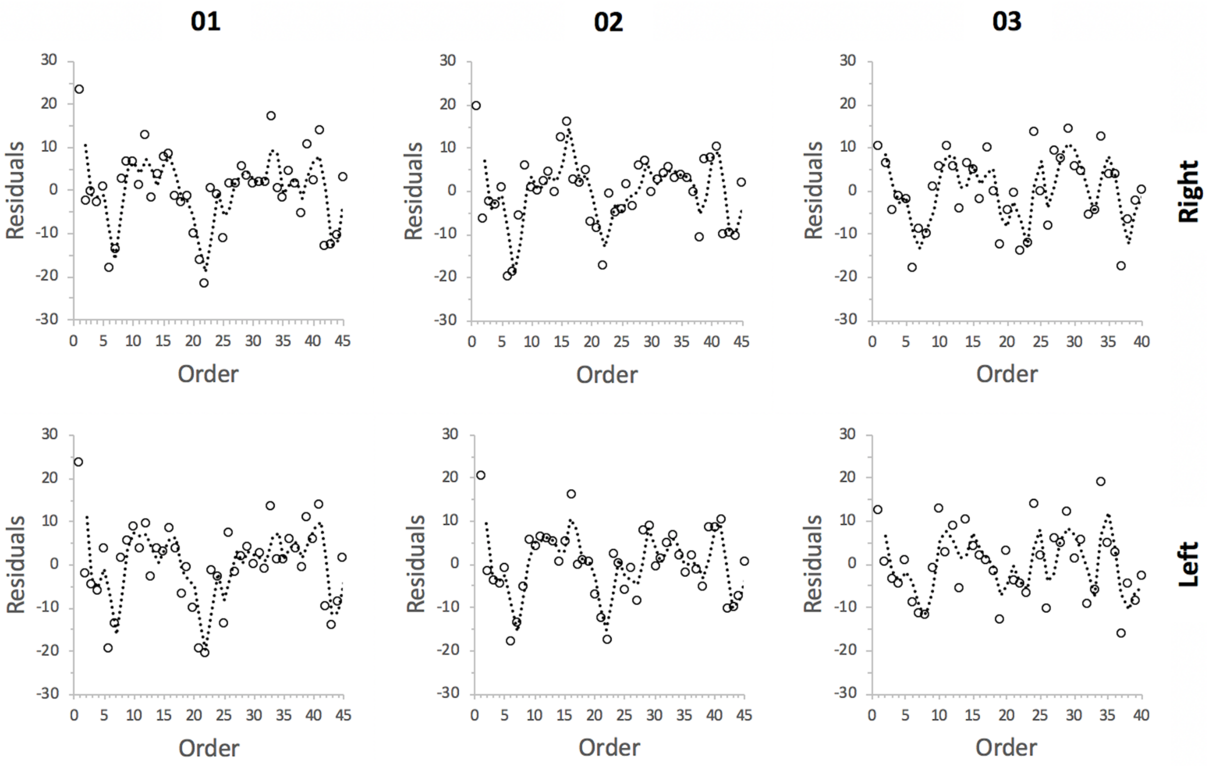

There is no discernible trend when plotting residuals (angle degrees) against the order in which data were collected (horse 1 - 45/40). Thus, the observations and associated error terms are considered independent satisfying the regression assumption.

D

Residuals vs age plots by side and tooth position

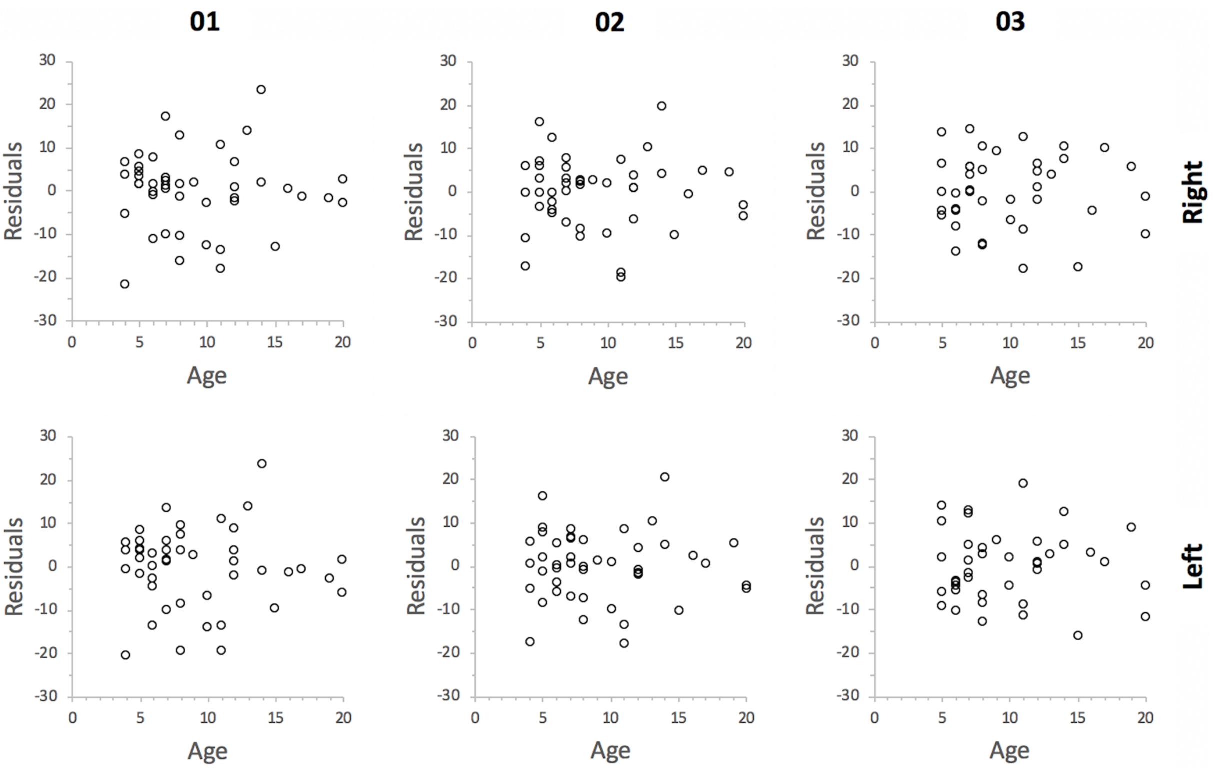

The residuals (angle degrees) bounce randomly around the zero-line. Hence the variation of residuals remains constant over the range of the explanatory variable (age) which indicates no influence of age (years) on parameter estimates.

E

Normal Q-Q-plots of residuals (observed IIA) by side and tooth position

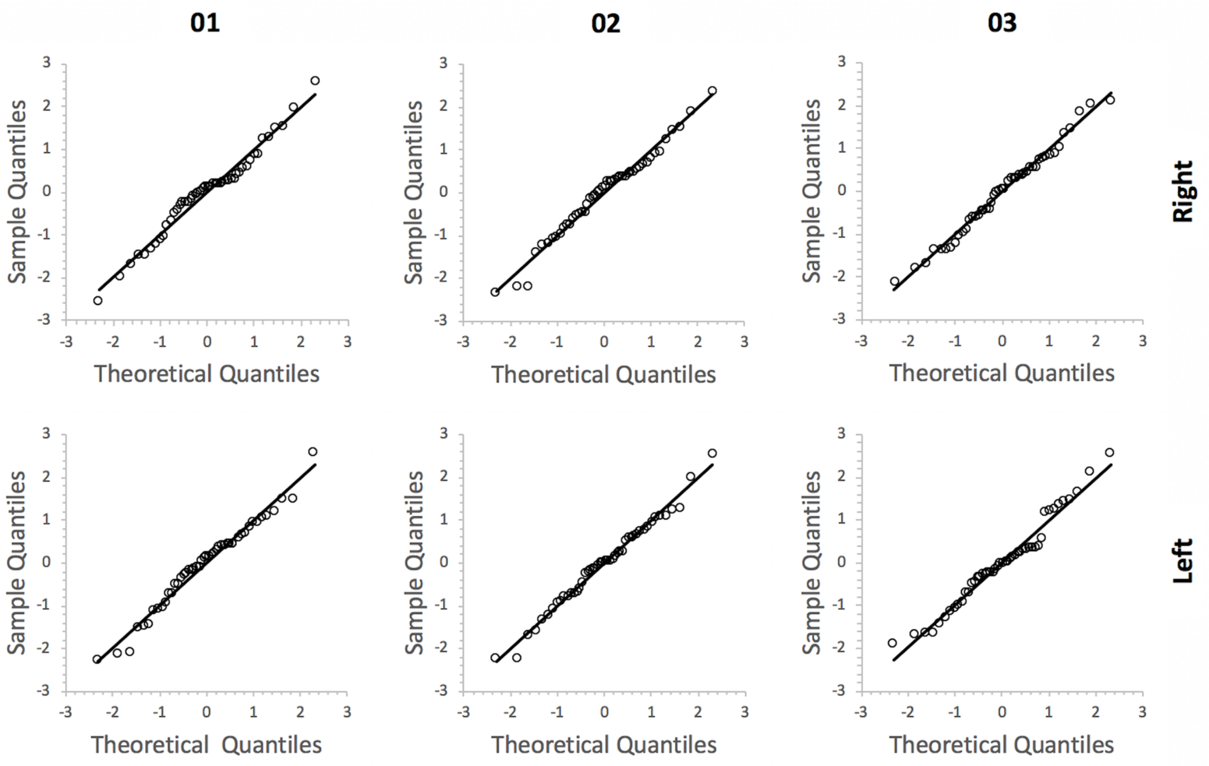

Plotting quantiles of sample residuals (sample quantiles) against quantiles, which were calculated from a standard normal distribution (theoretical quantiles) visually confirmed that there is no violation of the normal distribution of sample residuals from nonlinear regression analysis.

F

Overall normal Q-Q-plot and histogram of residuals (observed IIA)

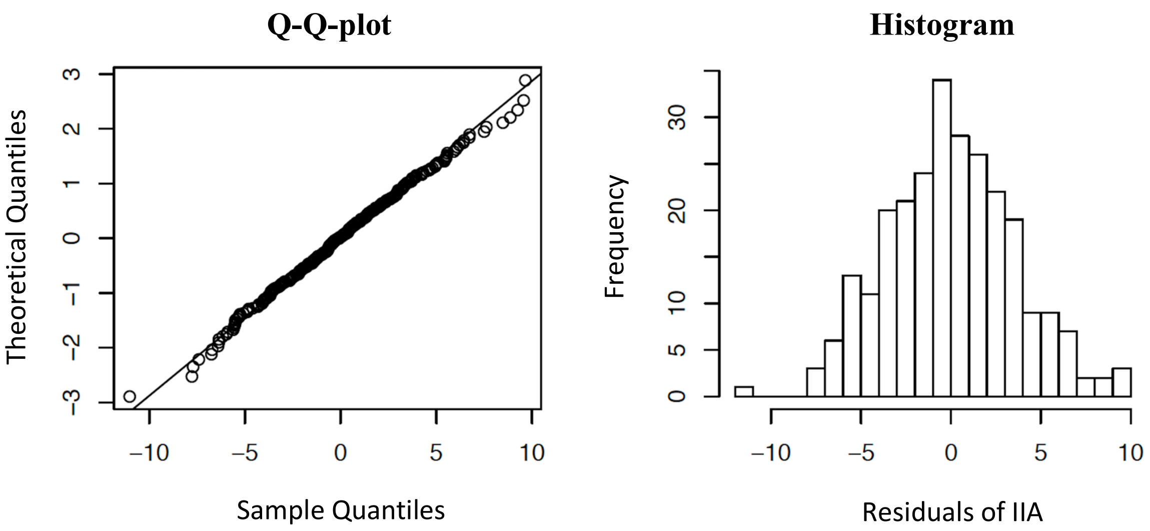

Plotting quantiles of sample residuals (sample quantiles) against quantiles, which were calculated from a standard normal distribution (theoretical quantiles) and the frequency distribution of residuals (histogram) visually confirmed that there is no violation of normal distribution of sample residuals from the GLME model. Model: IIA ~ Site + Age * Tooth position + (1 | horse ID).
