# Supplementary material for: Computed Tomography (CT)-Assisted 3D Cephalometry in Horses: Interincisal Angulation of Clinical Crowns
Source: Front Vet Sci. 2020 Jul 29;7:434. doi: 10.3389/fvets.2020.00434 (PMC7403475; doi:10.3389/fvets.2020.00434)
Supplement: Supplementary file 2 [file Table_1.pdf]

Suppl. Table 1. Randomised measurement order (A) and results of repeated IIA measures (B).

A

Horse	Age (y)		Measuring day									
			1	2	3	4	5	6	7	8	9	10
1	4	Measurement order (horse)	1	2	7	4	3	3	8	5	2	2
2	5		5	5	1	1	5	2	9	6	10	1
3	6		2	1	2	6	9	9	1	9	6	5
4	9		3	6	3	3	4	6	4	7	7	9
5	11		8	3	9	5	8	10	3	3	8	7
6	14		4	9	5	10	6	7	6	10	1	8
7	15		9	10	10	9	2	8	10	4	9	6
8	17		10	7	8	2	10	1	5	8	3	3
9	19		7	4	6	7	7	5	2	1	4	4
10	20		6	8	4	8	1	4	7	2	5	10

B

Horse	Opposing incisors	Measuring method	Repeated measures IIA (α)									
			1	2	3	4	5	6	7	8	9	10
1	01	LACC	153,5	154,4	152,8	153,3	153,5	153,7	153,1	155,2	153,8	155,3
	02	LACC	149,0	148,4	149,3	148,9	148,6	149,7	149,0	149,8	149,7	150,5
	03	LACC	123,0	123,5	123,5	123,0	123,6	122,7	123,5	118,1	122,0	122,4
	03	LPB*	-	-	-	-	-	-	-	-	-	-
2	01	LACC	147,9	146,4	148,6	147,2	148,0	147,7	147,7	147,7	148,0	148,3
	02	LACC	144,0	146,0	144,7	144,9	146,9	144,6	145,0	145,4	145,4	144,9
	03	LACC	148,0	150,2	148,1	148,3	149,9	149,7	149,0	148,1	148,6	149,2
	03	LPB	126,2	128,6	134,1	127,1	132,5	133,6	129,7	134,1	134,0	133,6
3	01	LACC	140,6	139,5	138,9	139,5	138,3	140,9	139,6	139,3	139,4	138,7
	02	LACC	137,7	135,6	136,1	136,3	135,2	135,3	136,1	135,4	135,9	134,3
	03	LACC	120,4	119,9	119,5	119,8	120,2	120,4	120,3	119,0	120,1	120,7
	03	LPB	111,6	113,0	113,2	113,0	112,5	109,7	112,4	109,8	110,2	115,0
4	01	LACC	131,6	131,7	131,2	131,7	130,5	132,0	131,7	131,3	131,6	132,5
	02	LACC	131,0	129,6	130,3	130,0	130,3	131,6	130,4	131,3	130,2	130,9
	03	LACC	123,1	123,5	124,6	123,5	124,0	124,7	123,8	124,8	124,4	123,8
	03	LPB	116,4	117,6	120,5	117,0	118,3	120,2	117,8	118,0	118,2	116,8
5	01	LACC	139,3	137,1	136,9	137,0	136,6	137,5	137,3	135,9	136,7	138,6
	02	LACC	132,9	132,0	131,7	132,1	131,4	131,0	131,4	130,3	131,4	130,8
	03	LACC	122,5	122,4	122,0	122,4	122,3	122,9	122,5	122,3	122,0	122,1
	03	LPB	126,6	128,3	128,5	127,9	128,3	131,3	128,1	130,2	129,8	128,2
6	01	LACC	146,8	148,4	147,3	147,2	145,4	147,7	147,4	146,3	147,0	149,0
	02	LACC	139,9	141,2	139,2	140,0	139,7	140,3	140,3	139,8	140,2	140,5
	03	LACC	115,6	118,0	115,1	115,5	115,1	117,2	116,7	116,1	116,0	115,2
	03	LPB	109,9	111,1	111,7	110,8	112,9	111,3	110,9	112,0	111,5	110,8
7	01	LACC	109,8	109,9	110,4	110,0	110,8	109,6	110,6	111,1	110,1	110,9
	02	LACC	109,8	108,4	108,7	109,1	108,0	109,2	108,9	110,4	108,8	109,7
	03	LACC	87,8	87,8	86,7	87,6	88,0	87,5	87,6	87,3	87,8	87,3
	03	LPB	71,7	70,4	74,7	71,0	72,5	71,2	72,9	72,2	72,0	72,3
8	01	LACC	121,0	121,0	121,1	121,1	121,1	121,5	121,4	120,1	121,2	120,1
	02	LACC	123,4	122,0	122,2	122,4	122,9	122,2	122,3	123,1	122,6	123,5
	03	LACC	112,2	112,4	114,3	112,8	114,7	116,0	113,8	113,6	113,5	114,3
	03	LPB	100,4	101,7	102,2	101,5	98,7	101,6	100,9	100,6	100,0	99,2
9	01	LACC	120,3	117,6	121,3	119,8	120,0	118,3	119,8	119,0	119,7	123,8
	02	LACC	123,9	120,8	120,2	120,2	121,3	119,9	120,9	119,6	121,0	121,1
	03	LACC	109,7	107,2	107,8	108,0	108,4	110,5	109,0	108,9	109,3	110,1
	03	LPB	98,3	95,8	98,6	98,0	99,0	100,6	98,8	95,4	98,9	97,4
10	01	LACC	119,6	117,0	119,2	119,1	117,2	118,6	119,4	119,6	119,5	119,6
	02	LACC	113,2	113,1	111,8	112,2	114,1	113,4	113,0	112,4	112,7	113,4
	03	LACC	100,3	102,1	101,7	101,7	102,6	103,6	102,4	98,9	101,4	103,5
	03	LPB	89,8	88,5	91,3	90,0	88,9	91,3	90,2	91,3	91,1	91,5

LACC, labial axis of cinical crown: newly described measuring method; LPB, lingual and palatinal border: reference method for corner incisors; * The short and irregular lingual and palatal surface of the deciduous upper jaw incisor clinical crown did not allow accurate definition of a clinical crown axis, hence determination of the IIA was not possible.
